# A Novel Informatics Tool to Detect Periprocedural Antibiotic Allergy Adverse Events for Near Real-time Surveillance to Support Audit and Feedback

**DOI:** 10.1001/jamanetworkopen.2023.13964

**Published:** 2023-05-17

**Authors:** Samira Reyes Dassum, Hillary J. Mull, Samuel Golenbock, Rebecca P. Lamkin, Isabella Epshtein, Marlena H. Shin, Judith M. Strymish, Kimberly G. Blumenthal, Kathryn Colborn, Westyn Branch-Elliman

**Affiliations:** 1 Department of Infectious Disease, Beth Israel Deaconess Medical Center, Boston, Massachusetts; 2 Center for Organization and Implementation Research, VA Boston Healthcare System, Boston, Massachusetts; 3 Department of Surgery, Boston University School of Medicine, Boston, Massachusetts; 4 Section of Infectious Disease, Department of Medicine, VA Boston Healthcare System, Boston, Massachusetts; 5 Division of Rheumatology, Allergy, and Immunology, Department of Medicine, Massachusetts General Hospital, Boston; 6 Department of Surgery, University of Colorado, Aurora; 7 Harvard Medical School, Boston, Massachusetts

## Abstract

**Importance:**

Standardized processes for identifying when allergic-type reactions occur and linking reactions to drug exposures are limited.

**Objective:**

To develop an informatics tool to improve detection of antibiotic allergic-type events.

**Design, Setting, and Participants:**

This retrospective cohort study was conducted from October 1, 2015, to September 30, 2019, with data analyzed between July 1, 2021, and January 31, 2022. The study was conducted across Veteran Affairs hospitals among patients who underwent cardiovascular implantable electronic device (CIED) procedures and received periprocedural antibiotic prophylaxis. The cohort was split into training and test cohorts, and cases were manually reviewed to determine presence of allergic-type reaction and its severity. Variables potentially indicative of allergic-type reactions were selected a priori and included allergies entered in the Veteran Affair’s Allergy Reaction Tracking (ART) system (either historical [reported] or observed), allergy diagnosis codes, medications administered to treat allergic reactions, and text searches of clinical notes for keywords and phrases indicative of a potential allergic-type reaction. A model to detect allergic-type reaction events was iteratively developed on the training cohort and then applied to the test cohort. Algorithm test characteristics were assessed.

**Exposure:**

Preprocedural and postprocedural prophylactic antibiotic administration.

**Main Outcomes and Measures:**

Antibiotic allergic-type reactions.

**Results:**

The cohort of 36 344 patients included 34 703 CIED procedures with antibiotic exposures (mean [SD] age, 72 [10] years; 34 008 [98%] male patients); median duration of postprocedural prophylaxis was 4 days (IQR, 2-7 days; maximum, 45 days). The final algorithm included 7 variables: entries in the Veteran Affair’s hospitals ART, either historic (odds ratio [OR], 42.37; 95% CI, 11.33-158.43) or observed (OR, 175.10; 95% CI, 44.84-683.76); PheCodes for “symptoms affecting skin” (OR, 8.49; 95% CI, 1.90-37.82), “urticaria” (OR, 7.01; 95% CI, 1.76-27.89), and “allergy or adverse event to an antibiotic” (OR, 11.84, 95% CI, 2.88-48.69); keyword detection in clinical notes (OR, 3.21; 95% CI, 1.27-8.08); and antihistamine administration alone or in combination (OR, 6.51; 95% CI, 1.90-22.30). In the final model, antibiotic allergic-type reactions were identified with an estimated probability of 30% or more; positive predictive value was 61% (95% CI, 45%-76%); and sensitivity was 87% (95% CI, 70%-96%).

**Conclusions and Relevance:**

In this retrospective cohort study of patients receiving periprocedural antibiotic prophylaxis, an algorithm with a high sensitivity to detect incident antibiotic allergic-type reactions that can be used to provide clinician feedback about antibiotic harms from unnecessarily prolonged antibiotic exposures was developed.

## Key Points

**Question:**

Can an informatics tool improve detection of antibiotic allergic-type events?

**Findings:**

In this cohort study of 36 344 patients who underwent cardiovascular implantable electronic device procedures, an electronic algorithm with high positive predictive value was developed that can be used to measure the occurrence of periprocedural antibiotic allergic-type events in near real-time. Most antibiotic allergic-type reactions were moderate in severity.

**Meaning:**

The algorithm developed in this study could be used to detect when antibiotic allergic-type reactions occur to provide direct clinician feedback about harms.

## Teaser

This cohort study describes the development of an informatics tool to improve detection of periprocedural antibiotic allergy adverse events.

## Introduction

Adverse events associated with antibiotic use include *Clostridioides difficile* infection, acute kidney failure, and immunologically mediated hypersensitivity reactions.^1,2,3^ Antibiotics are among the most common drugs associated with hypersensitivity reactions in hospitalized patients.^4^ The downstream effects posed by an antibiotic allergy include increased length of stay and health care costs, use of broad-spectrum antibiotics, and mortality.^5,6,7^

Antibiotics that most commonly cause hypersensitivity reactions (β lactams, vancomycin, sulfonamides) are the same drugs commonly prescribed for surgical site infection prophylaxis.^8,9^ Despite clinical guidelines recommending against extending duration of prophylaxis following skin closure, prolonged postprocedural antibiotics are commonly prescribed in some clinical settings, increasing the risk of adverse events, including antibiotic allergic-type reactions.^10,11,12,13^ However, the rate of antibiotic-related adverse reactions and allergy events that occur in this context is unknown.

There is no current process for antibiotic drug reaction surveillance. Lack of adverse event detection prevents feedback to clinicians about direct patient harms of inappropriate antibiotic use. Thus, the aims of this retrospective, multicenter national cohort study were (1) to develop an electronic tool using structured and unstructured data elements available within the Veterans Affairs (VA) electronic health record (EHR) to identify incident antibiotic allergic-type reactions and (2) to characterize the nature and severity of these reactions.

## Methods

### Ethical Considerations

The VA Boston Healthcare System institutional review board approved this as an exempt study with a waiver of informed consent given the retrospective and observational nature of the study. The study followed the Strengthening the Reporting of Observational Studies in Epidemiology (STROBE) reporting guidelines for cohort studies.

### Background and Overview

This nationwide, retrospective cohort study was conducted as part of a larger quasi-experimental study to leverage audit and feedback about antibiotic adverse events and promote deimplementation of periprocedural antibiotic use.^14^ Cardiac electrophysiology procedures were selected due to high-rates of guideline-discordant prolonged postprocedural prophylaxis in this area, often lasting for as long as 14 days following skin closure.^10,11,15,16^

### Setting, Study Population, and Data Sources

The patient population included all cardiovascular implantable electronic device (CIED) procedures (implantations and revisions of permanent pacemakers, implantable cardioverter defibrillators [ICDs], biventricular pacemaker ICDs, Watchman device placements) performed at Veterans Health Administration (VHA) facilities from October 1, 2015, to September 30, 2019. Data were analyzed between August 1, 2021, and January 31, 2022. Individual CIED procedures were included in the study if they had received 1 or more antibiotic(s) within 1 day before or after their procedure.

EHR data were extracted from the VA Corporate Data Warehouse using SQL Server Management Studio 18 (Microsoft Corp). Data sources accessed include administrative files (eg, hospital visit dates, *International Statistical Classification of Diseases and Related Health Problems, Tenth Revision *[*ICD-10*] diagnosis codes, procedure codes, patient characteristics), structured tables (orders and dispensed medications), and unstructured clinical text notes. Duration of antibiotic exposure following CIED procedure was calculated based on the number of days of documented antibiotic administrations (inpatient data) and/or the number of days of antibiotic fills (outpatient exposures). Patient comorbidities were extracted using the Agency for Healthcare Research and Quality Elixhauser Comorbidity Software.^17^

### Potential Elements of Allergic-Type Reactions

Variables potentially indicative of an allergic-type reaction event were selected a priori based on clinical experience, knowledge of VA databases, and a literature review.^2,18,19,20,21^ These included entries in the VA’s Allergy Reaction Tracking (ART) system (allergies entered into the EHR, either historical [reported] or observed), allergy diagnosis codes (eg, rash, skin eruption due to medications, urticaria), medications administered to treat allergic-type reactions (eg, antihistamines, corticosteroids, epinephrine; intravenous or oral), text searches of clinical notes for keywords and phrases indicative of a potential allergic-type reaction, and consults to allergy and dermatology (eTable 1 in Supplement 1). Data containing these variables were extracted between the procedure date and 45 days thereafter.

### Medical Record Review Sampling Strategy

The patient population was divided into training and testing cohorts. Cases were then selected from the training cohort to undergo manual review (eFigure 1 in Supplement 1). In the training medical record review sample, cases were selected to ensure sampling of different variables and negative cases. After model training, a second round of medical record review from a sample of the testing cohort was undertaken. Cases were selected for medical record review with a planned weighted random sampling procedure based on a positive predictive value (PPV) cutoffs estimated from the model developed using the training medical record review sample. The primary case reviewer (S.R.D.) was blinded to the algorithm determination for the purposes of model testing.

### Medical Record Review Process and Clinical Definitions

The manual medical record review process included a standardized review tool.^22^ Manual review of sample cases with none, 1, or more variables during the period from CIED procedure date (eg, first day of antibiotic exposure) and the subsequent 45-day window period was completed. The surveillance period for antibiotic-associated allergic-type reactions was a maximum of 10 days after the last date of antibiotic exposure, which was calculated based on medication administration data, days of antibiotic dispensed, or both. Manually abstracted data included procedure date, adverse event date and type (eg, allergic-type reaction, adverse reaction, and other), triggered variable, culprit antibiotic, reaction noted, treatment received, and change in level of care (eg, outpatient to inpatient, inpatient to intensive care unit). Each event was then classified into allergic-type reaction, adverse reaction, or other or no event occurrence. Allergic-type reactions were defined as allergic signs or symptoms that could be immune-mediated hypersensitivity reactions, such as rash, hives, or anaphylaxis occurring after antibiotic exposure.^2^ Adverse reaction was defined as a nonimmunologically mediated undesired response to an antibiotic (eg, nausea, vomiting, diarrhea).^2^ Among patients who experienced an allergic-type reaction, severity of allergies was classified as mild, defined as not requiring medication or change in level of care; moderate, defined as requiring medication but no change in level of care; and severe, defined as requiring medication and change in level of care or intubation.^23,24,25^

### Statistical Analysis

The primary outcome was manually adjudicated antibiotic allergic-type reaction. Antibiotic allergic-type reaction was modeled using the training medical record review sample. An initial set of factors associated with allergic-type reaction from 4 a priori allergy detection categories (listed previously) were selected using a generalized linear model with a logit link (via the SAS Procedure HPGENSELECT), with a least absolute shrinkage and selection operator (LASSO) regression analysis for variable selection (SAS Enterprise Guide version 8.2 [SAS Institute]). Potential factors were iteratively updated to increase estimated probability of identifying true antibiotic allergic-type reactions, eg, by adjusting keyword searches and using diagnosis phenotype codes (PheCodes)^20,21,26^ rather than *ICD-10* codes (eTables 2-4 in Supplement 1).

Different iterations of the model were subsequently tested using logistic regression to estimate odds ratios (ORs) for different variables and estimate the probability of an antibiotic allergic-type reaction. The final version of the model was selected based on the estimated probability cutoff with the highest positive predictive value (PPV) and C statistic. Unadjusted and adjusted (full model) ORs for the outcome and the receiver operating characteristic were calculated in the training data set using the final model.

After selecting a predictive model using the training medical record review sample, logistic regression coefficients were applied to the testing cohort to obtain estimated probabilities that an allergic-type reaction had occurred. The test medical record review sample was selected according to a predetermined but arbitrarily selected estimated probability distribution to oversample high-probability cases. After reviewing the sampled procedures from the testing cohort, we assessed criterion validity (PPV, sensitivity) of the final model and compared it with the model performance in the training medical record review sample. Criterion validity of the algorithm was assessed with criterion-standard manual medical record review.^27^

## Results

### Patient Sample

A total of 36 344 patients underwent CIED procedures during the study period, and 34 703 (96%) had a periprocedural antibiotic exposure and were included in the study. The median duration of postprocedural antibiotic prophylaxis in the whole cohort was 4 days (IQR, 2-7 days; maximum, 45 days). The total population was divided into a training data set (17 350 participants) and a testing data set (17 353 participants). Most included patients were male (34 008 [98%]) and White (26 576 [77%]). The mean (SD) age was 72 (10) years (Table 1).^21,22,26^

**Table 1.** Characteristics of Patients Undergoing Cardiac Device Implantation in the Training Cohort and Training Medical Record Review Sample

Characteristic	Patients, No. (%)				*P* value^a^
	Training cohort (n = 17 350)	Training medical record review sample			
		Allergic reaction (n = 96)	No allergic reaction (n = 335)		
**Patient characteristics**					
Age at procedure, mean (SD)	72.1 (10.4)	71.3 (12.0)		72.1 (10.3)	.55
Sex					
Female	373 (2.1)	3 (3.1)		9 (2.7)	.73
Male	16 977 (97.9)	93 (96.9)		326 (97.3)	
Race and ethnicity					
Black	2661 (15.4)	6 (6.3)		48 (14.3)	.04
White	13,267 (76.5)	77 (80.2)		260 (77.6)	
Unknown or other^b^	1422 (8.2)	13 (13.5)		27 (8.1)	
Select comorbidities					
Hypertension	13 055 (75.2)	71 (74)		261 (77.9)	.42
Diabetes	7387 (42.6)	46 (47.9)		146 (43.6)	.45
Heart failure	3918 (22.6)	20 (20.8)		103 (30.7)	.06
Malignant neoplasm	1262 (7.3)	7 (7.3)		24 (7.2)	.97
Prior year antibiotic allergy	203 (1.2)	4 (4.2)		20 (6.0)	.50
**Variable detection rate**					
ART (observed)^c^	48 (0.3)	60 (62.5)		19 (5.7)	<.001
ART (historical)^c^	83 (0.5)	23 (24)		57 (17)	.12
Clinical note (keyword: symptom and medication)	1510 (8.7)	51 (53.1)		85 (25.4)	<.001
Antihistamine	500 (2.9)	35 (36.5)		54 (16.1)	<.001
Antihistamine with steroid	68 (0.4)	15 (15.6)		16 (4.8)	<.001
Steroid	352 (2.0)	25 (26)		31 (9.3)	<.001
Epinephrine	94 (0.5)	1 (1)		11 (3.3)	.48
New allergy *ICD* code (45 d postprocedure)	229 (1.3)	40 (41.7)		61 (18.2)	<.001
PheCode: contact dermatitis^d^	18 (0.1)	7 (7.3)		14 (4.2)	.28
PheCode: anaphylactic shock NOS	3 (0)	1 (1)		2 (0.6)	.53
PheCode: urticaria	25 (0.1)	13 (13.5)		11 (3.3)	<.001
PheCode: allergies, other	18 (0.1)	2 (2.1)		17 (5.1)	.27
PheCode: AE antibiotic and penicillin	39 (0.2)	19 (19.8)		17 (5.1)	<.001
PheCode: AE nonspecific drug	7 (0)	0 (0)		7 (2.1)	.36
PheCode: rash and skin erupt	151 (0.9)	17 (17.7)		11 (3.3)	<.001
ART (observed) with note keyword	28 (0.2)	30 (31.3)		11 (3.3)	<.001
ART (observed) with medication	20 (0.1)	28 (29.2)		5 (1.5)	<.001
ART (observed) with PheCode: AE antibiotic and penicillin	15 (0.1)	22(22.9)		4 (1.2)	<.001

Abbreviations: AE, adverse event; ART, Adverse Reaction Tracking; *ICD*, *International Classification of Diseases*; NOS, not otherwise specified.

^a^ *P* value between manually adjudicated allergic reaction vs cases without allergic reaction: χ^2^ for categorical variables, *t* test for continuous variables, Fisher exact test for cell counts less than 5.

^b^ Other races included American Indian or Alaska Native; Asian; Native Hawaiian or other Pacific Islander; and race unknown. Other ethnicities include Hispanic or Latino and not Hispanic or Latino.

^c^ An allergy that is documented in the allergy module of the electronic medical record.

^d^ PheCode: Diagnosis phenotype taken from PheWas Database.^20,21,26^

### Allergic-Type Reaction Algorithm Development

The algorithm training cohort included 17 350 procedures, of which 311 underwent manual review (training medical record review sample). In the training sample, the initial set of variables selected by the LASSO included observed ART flag; keywords documented in clinical notes; corticosteroid receipt; new *ICD-10* code; and PheCodes for urticaria, adverse event to antibiotic and penicillin, and rash and skin eruption (Table 2). Prespecified combinations significantly associated with identification of an allergic-type reaction included observed ART flag and keyword in note; observed ART flag and anti-allergy medication administered; and observed ART flag and PheCode for adverse event to an antibiotic and penicillin. Variables evaluated as potential factors that did not have discriminative utility included allergy and dermatology consultations; epinephrine; and PheCodes for contact dermatitis, anaphylactic shock, allergies, and adverse event associated with nonspecific drug.

**Table 2.** Antibiotic Allergy Detection Algorithm Logistic Regression Results of the Final Model Among Training Cohort

Variables	OR (95% CI)	
	Unadjusted	Adjusted
ART (historical)	3.43 (1.71-6.92)	42.37 (11.33-158.43)
ART (observed)	42.37 (17.29-103-85)	175.10 (44.84-683.76)
PheCode: symptoms affecting the skin	6.06 (2.33-15.78)	8.49 (1.90-37.82)
PheCode: urticaria	4.35 (1.79-10.56)	7.01 (1.76-27.89)
PheCode: allergy or adverse event to antibiotic	4.14 (1.84-9.34)	11.84 (2.88-48.69)
Keyword searches	3.70 (2.10-6.52)	3.21 (1.27-8.08)
Corticosteroid receipt	2.98 (1.53-5.83)	6.51 (1.90-22.30)

Abbreviations: ART, Adverse Reaction Tracking; OR, odds ratio.

The final model included 7 variables indicative of an antibiotic allergic-type reaction during the 45-day period following antibiotic exposure (C statistic = 0.95; PPV, 77%; 95% CI, 66%-86%) (Table 2 and Table 3; eFigure 2 in Supplement 1). The variables retained in the final model included: entries into ART system, either historic (OR, 42.37; 95% CI, 11.33-158.43) or observed (OR, 175.10; 95% CI, 44.84-683.76); PheCodes for “symptoms affecting the skin” (OR, 8.49; 95% CI, 1.90-37.82), “urticaria” (OR, 7.01; 95% CI, 1.76-27.89), and “allergy or adverse event to an antibiotic” (OR, 11.84; 95% CI, 2.88-48.69); keyword detection in clinical notes (OR, 3.21; 95% CI, 1.27-8.08); and antihistamine administration alone or in combination (OR, 6.51; 95% CI, 1.90-22.30). Based on the area under the curve of the model, a PPV cutoff of 30% was selected to optimize sensitivity and specificity.

**Table 3.** Distribution of Probability of Antibiotic Allergic Reaction and PPV Results

Probability range, %	Training sample (n = 311), No. (%)	Allergic reactions	PPV (95% CI), %	No. (%)			PPV, %
				Test cohort (n = 17 353)	Test sample (n = 120)	Allergic-type reactions	
≤10	190 (61.1)	1 (1.5)	NA	16,996 (97.9)	40 (44.9)	0	NA
11-20	22 (7.1)	3 (4.6)		183 (1.05)	0	0	
21-30	29 (9.3)	7 (10.8)		80 (0.46)	36 (40.4)	4 (12.90)	
31-40	2 (0.6)	0	77 (66-86)	0	0	0	61 (45-76)
41-50	9 (2.9)	5 (7.7)		8 (0.05)	2 (2.3)	0	
51-60	13 (4.2)	9 (13.9)		31 (0.18)	18 (20.2)	10 (32.3)	
61-70	4 (1.3)	4 (6.2)		4 (0.02)	2 (6.5)	2 (6.5)	
71-80	4 (1.3)	4 (6.2)		3 (0.02)	0	0	
81-90	16 (5.1)	13 (20)		18 (0.10)	7 (7.9)	3 (9.7)	
>90	22 (7.1)	19 (29.2)		30 (0.17)	15 (16.8)	12 (38.7)	

Abbreviations: NA, not applicable; PPV, positive predictive value.

### Model Testing

The logistic regression model from the training cohort was applied to the testing cohort of 17 353 participants. A total of 17 259 cases (99%) fell under the PPV threshold for detecting potential allergic-type reactions; 120 cases underwent manual review (testing medical record review sample). In this sample, 87% of allergic-type reactions (27 cases) fell into the high probability category. The PPV of the final model in the test data set was 61% (95% CI, 45%-76%), and sensitivity was 87% (95% CI, 70%-96%). The ART flag alone had lower sensitivity but similar PPV as the more complex model (sensitivity, 77%; 95% CI, 59%-90%; specificity, 87%; 95% CI, 78%-93%; PPV, 66.7%; 95% CI, 49%-81%).

### Nature and Severity of Antibiotic Allergic-Type Reactions

Allergic-type reactions by class of antibiotic, intervention administered, and severity classification are presented in Table 4.^24,25^ Among cases with allergic-type reactions, the median (IQR) duration of postprocedure exposure was 5 (2-7) days; the same time frame when most events (75 [72%]) occurred. The most frequently administered antibiotics associated with allergic-type reactions included cephalosporins (40%) and vancomycin (23%), the same antibiotics most frequently administered for periprocedural prophylaxis.

**Table 4.** Nature and Severity of Antibiotic-Associated Allergic-Type Reactions

Antibiotic	Allergic-type reaction No. (%)	Intervention, No.			Allergic-type reaction severity, No.^a^			
		Antihistamine	Corticosteroid	Epinephrine (intravenous)	Mild	Moderate	Severe	Intubation or ICU
Cephalosporins	42 (40)	21	16	1	15	25	6	NA
Vancomycin	24 (23)	8	10	NA	13	14	NA	NA
Tetracycline	9 (9)	4	3	NA	5	1	5	NA
Penicillin	6 (6)	3	2	NA	3	1	2	NA
Sulfa	5 (5)	2	3	NA	1	2	2	NA
Clindamycin	8 (8)	4	2	NA	2	7	1	NA
Daptomycin	3 (3)	1	NA	NA	2	NA	1	NA
Fluoroquinolone	3 (3)	3	3	NA	NA	2	1	NA
Carbapenem	2 (2)	NA	2	1	1	NA	1	NA
Macrolide	2 (2)	NA	NA	1	1	NA	1	1
Total^b^	104 (100)	46	41	3	43	52	20	1

Abbreviations: ICU, intensive care unit; NA, not applicable.

^a^ Severity scale based on Ring and Messmer and Brown Allergy severity scales.^24,25^

^b^ Total number of drug exposures, which occurred in a total of 102 patients.

Among the manually reviewed cases from the whole cohort, 104 antibiotic allergic-type reactions occurred following 102 exposures (due to receipt of multiple different types of antibiotics, resulting in multiple allergy labels). Of these, 96 were due to antibiotics administered for periprocedural prophylaxis, and 81 allergic-type reactions (84%) were attributed to antibiotics administered for longer than 24 hours following skin closure. Of the 96 antibiotic allergic-type reactions attributed to periprocedural prophylaxis, 60 (63%) had an observed ART flag, 23 (24%) had a historic ART flag, and 30 of 96 cases (38%) did not have the allergy listed in the electronic medical record.

Most antibiotic allergic-type reactions detected in the inpatient setting and were moderate in severity, requiring administration of antihistamine, corticosteroid, or intravenous epinephrine or a combination but did not require change in level of care. Most cases that required change in level of care (eg, severe events) were outpatients treated in the emergency department. Intubation or intensive care unit was not required for any of the reactions with cephalosporins or vancomycin. One patient who presented with pneumonia and received azithromycin developed anaphylactic shock, was intubated in the emergency department, and was admitted into the intensive care unit.

## Discussion

Antibiotics are one of the most common causes of medication adverse events.^6,7^ Despite this, guideline-discordant prolonged prophylaxis lasting longer than 24 hours after skin closure remains common in some surgical and procedural settings, including the cardiac electrophysiology laboratory.^11,12,28^ Identification of allergic-type reactions in near real-time creates an opportunity for audit and feedback to clinicians about patient harms of antibiotic prescribing and an opportunity to encourage adoption of evidence-based practices regarding antibiotic administration for postprocedural prophylaxis. Systematic surveillance may be particularly important in outpatient and procedural settings, as many events may occur after discharge and thus clinicians remain unaware of preventable harm caused by inappropriate antibiotic administration. The algorithm developed and validated in this study helps close this gap and can potentially be used to enhance identification of allergic-type reactions and improve quality and safety of medical care.

Most allergic-type reactions occurred shortly after antibiotic exposure (within 1 week of the procedure and during the period of ongoing postprocedural prophylaxis) and were attributed to antibiotics administered for guideline-discordant, prolonged prophylaxis.^10,16^ These findings highlight the importance of perioperative antibiotic stewardship to limit harms of unnecessary antibiotic exposures and the need for innovative strategies to provide audit and feedback to clinicians about patient harm caused by unnecessary antibiotic exposures.

In our study, the rate of antibiotic allergic-type reactions occurring with prolonged postprocedural antibiotic use was higher than previously reported rates (0.9% for cefazolin and 1.1% for vancomycin).^29,30^ The higher rate in our study is likely due to a combination of the duration of prophylaxis and exposure to different agents for prophylaxis (eg, vancomycin and/or cefazolin periprocedure, followed by an outpatient oral antibiotic, such as cephalexin or doxycycline). Importantly, given these procedures were performed among individuals without an underlying infection, we were able to identify allergic-type reactions without the need to distinguish allergic reactions from other entities that cause rash, such as viral exanthems.

This study adds to previous literature about the harms of extending prophylaxis beyond the guideline-recommended period and can be used in future investigations to consider a composite adverse event risk metric from antibiotic exposure, rather than considering each harm individually. Antibiotic-associated adverse events when considered individually (eg, incidence of *C difficile* infection following a single exposure) are rare; however, when a broader array of outcomes is considered, incidence of overall harm to the individual patient increases and contributes more substantially to adverse patient outcomes. More research is needed to develop antibiotic-use harm metrics that fully capture all harms caused by antibiotics to better inform and adapt clinical practice.

A simple model that included only the ART flag had lower sensitivity but similar PPV as the more complex algorithm that included both structured and unstructured data. Thus, depending on the goals of the surveillance program and available information technology resources to support stewardship initiatives, use of the ART flag alone without the added complexity of text-note searching is a viable alternative strategy that may balance accuracy of event detection with feasibility.

### Limitations

This study has limitations. The algorithm was developed to identify cases with a high probability of having an antibiotic allergic-type reaction but does not replace clinician input and clinical review. However, from a practical standpoint, it can substantially reduce medical record review burden, as most cases (>99%) fell below the probability threshold that would require manual adjudication. Ideally, the algorithm would assist with attributing an allergic-type reaction to a specific exposure. However, in our cohort, patients received more than 1 antibiotic during the specified period, and pinpointing the causative agent was not possible without manual adjudication.

The algorithm primarily detected allergic-type reactions of moderate severity. This may be due to moderate severity allergic-type reactions being more common, or due to inherent features of the algorithm. Cutaneous manifestations were the most commonly reported allergic-type reactions, and these are easily treated in the inpatient setting with diphenhydramine or steroids, which were included in the algorithm.^8^ Mild reactions that did not require medical intervention are less likely to be detected, as they are less likely to require medical care and therefore documentation in the EHR. However, they are likely less important from a clinical standpoint. Severe reactions were also less likely to be identified, likely because they occur less frequently.^8,31^ However, it is possible that severe reactions are more common than was identified, as patients with severe reactions may be taken to the nearest emergency department, not necessarily to a VA medical center, and therefore, the events might not be documented in the VA EHR.

Our study is subject to the limitations of documentation in the EHR and sampling methodology. False-positive variable detection related to historic *ICD-10-DM* codes (eg, for a history of penicillin allergy) were copied forward in EHR documentation, even though no penicillin exposure occurred during the incident admission. Missing data due to inability to detect adverse reactions that are treated outside the VA is another limitation and may have affected the number of cases identified and the true severity distribution of antibiotic allergic-type reactions. Our validation method focused on oversampling high probability cases, which may have led to an underestimation of the true incidence of adverse events due to limited review of potentially false-negative cases. It is not clear how well the tool would perform outside of the closed VA health care system or other EHRs or applied to other clinical settings or more complex or prolonged surgical procedures.

Another limitation is that this study was conducted within the VA health care system, and the cohort included predominantly older White male patients. Drug allergies are much more commonly reported in female than male patients^32^; thus, the absolute incidence of allergic-type reactions may be underestimated in this population relative to populations with more women. Additionally, manual review was completed on a relatively small sample of the total cohort, and by a single adjudicator.

## Conclusions

In this cohort study, we developed and validated an informatics tool using 7 variables composed of structured and unstructured data for the detection of antibiotic allergic-type reactions. This model had a good PPV for detecting antibiotic allergic-type reactions and can be operationalized to provide clinicians with real-time audit and feedback about when allergic-type reactions occur to support efforts to reduce inappropriate and unnecessary antibiotic exposures that increase patient-level harm without improving outcomes.

## Notes

Supplement 1. **eTable 1.** Candidate Algorithm Variable Definitions **eTable 2.** *ICD-10-CM* Codes and Associated PheCodes for Possible Adverse Reaction to Antibiotic Medication **eTable 3.** Keywords Used to Search Electronic Health Record Text Notes **eTable 4.** Iterative Changes to the Algorithm **eFigure 1.** Sampling Strategy for Training and Testing Data Sets **eFigure 2.** Receiver Operating Curve for Model

Supplement 2.
Data Sharing Statement

## References

[1] Branch-Elliman W, O’Brien W, Strymish J, Itani K, Wyatt C, Gupta K. Association of duration and type of surgical prophylaxis with antimicrobial-associated adverse events. JAMA Surg. 2019;154(7):590-598. doi:10.1001/jamasurg.2019.0569 31017647 PMC6487902

[2] Blumenthal KG, Wolfson AR, Li Y, . Allergic reactions captured by voluntary reporting. J Patient Saf. 2021;17(8):e1595-e1604. doi:10.1097/PTS.0000000000000568 30720546 PMC6669104

[3] Asundi A, Stanislawski M, Mehta P, . Prolonged antimicrobial prophylaxis following cardiac device procedures increases preventable harm: insights from the VA CART program. Infect Control Hosp Epidemiol. 2018;39(9):1030-1036. doi:10.1017/ice.2018.170 30226128

[4] Thong BYH, Tan TC. Epidemiology and risk factors for drug allergy. Br J Clin Pharmacol. 2011;71(5):684-700. doi:10.1111/j.1365-2125.2010.03774.x 21480948 PMC3093074

[5] Blumenthal KG, Lu N, Zhang Y, Li Y, Walensky RP, Choi HK. Risk of meticillin resistant *Staphylococcus aureus* and *Clostridium difficile* in patients with a documented penicillin allergy: population based matched cohort study. BMJ. 2018;361:k2400. doi:10.1136/bmj.k2400 29950489 PMC6019853

[6] MacFadden DR, LaDelfa A, Leen J, . Impact of reported beta-lactam allergy on inpatient outcomes: a multicenter prospective cohort study. Clin Infect Dis. 2016;63(7):904-910. doi:10.1093/cid/ciw462 27402820

[7] Blumenthal KG, Lu N, Zhang Y, Walensky RP, Choi HK. Recorded penicillin allergy and risk of mortality: a population-based matched cohort study. J Gen Intern Med. 2019;34(9):1685-1687. doi:10.1007/s11606-019-04991-y 31011962 PMC6712108

[8] Blumenthal KG, Peter JG, Trubiano JA, Phillips EJ. Antibiotic allergy. Lancet. 2019;393(10167):183-198. doi:10.1016/S0140-6736(18)32218-9 30558872 PMC6563335

[9] Kabulski GM, Northup A, Wiggins BS. Postoperative antibiotic prophylaxis following cardiac implantable electronic device placement. J Innov Card Rhythm Manag. 2019;10(8):3777-3784. doi:10.19102/icrm.2019.100804 32477744 PMC7252755

[10] Bratzler DW, Dellinger EP, Olsen KM, ; American Society of Health-System Pharmacists; Infectious Disease Society of America; Surgical Infection Society; Society for Healthcare Epidemiology of America. Clinical practice guidelines for antimicrobial prophylaxis in surgery. Am J Health Syst Pharm. 2013;70(3):195-283. doi:10.2146/ajhp120568 23327981

[11] Mehrotra P, Gupta K, Strymish J, . Implementation of infection prevention and antimicrobial stewardship in cardiac electrophysiology laboratories: results from the SHEA Research Network. Infect Control Hosp Epidemiol. 2017;38(4):496-498. doi:10.1017/ice.2016.309 28103958 PMC5550107

[12] Branch-Elliman W, Stanislawski M, Strymish J, . Cardiac electrophysiology laboratories: a potential target for antimicrobial stewardship and quality improvement? Infect Control Hosp Epidemiol. 2016;37(9):1005-1011. doi:10.1017/ice.2016.116 27322021

[13] Uslan DZ, Gleva MJ, Warren DK, . Cardiovascular implantable electronic device replacement infections and prevention: results from the REPLACE Registry. Pacing Clin Electrophysiol. 2012;35(1):81-87. doi:10.1111/j.1540-8159.2011.03257.x 22077194

[14] Branch-Elliman W, Lamkin R, Shin M, . Promoting de-implementation of inappropriate antimicrobial use in cardiac device procedures by expanding audit and feedback: protocol for hybrid III type effectiveness/implementation quasi-experimental study. Implement Sci. 2022;17(1):12. doi:10.1186/s13012-022-01186-8 35093104 PMC8800400

[15] Blomstrom-Lundqvist C, Ostrowska B. Prevention of cardiac implantable electronic device infections: guidelines and conventional prophylaxis. Europace. 2021;23(suppl 4):iv11-iv19. doi:10.1093/europace/euab071 34037227 PMC8221047

[16] Berríos-Torres SI, Umscheid CA, Bratzler DW, ; Healthcare Infection Control Practices Advisory Committee. Centers for Disease Control and Prevention guideline for the prevention of surgical site infection, 2017. JAMA Surg. 2017;152(8):784-791. doi:10.1001/jamasurg.2017.0904 28467526

[17] Agency for Healthcare Research and Quality. Elixhauser comorbidity software refined for *ICD-10-CM*. Accessed June 20, 2022. https://www.hcup-us.ahrq.gov/toolssoftware/comorbidityicd10/comorbidity_icd10.jsp

[18] Saff RR, Li Y, Santhanakrishnan N, . Identification of inpatient allergic drug reactions using *ICD-9-CM* codes. J Allergy Clin Immunol Pract. 2019;7(1):259-264.e1. doi:10.1016/j.jaip.2018.07.022 30075337 PMC7213838

[19] Yang J, Wang L, Phadke NA, . Development and validation of a deep learning model for detection of allergic reactions using safety event reports across hospitals. JAMA Netw Open. 2020;3(11):e2022836. doi:10.1001/jamanetworkopen.2020.22836 33196805 PMC7670315

[20] Wei WQ, Bastarache LA, Carroll RJ, . Evaluating PheCodes, clinical classification software, and *ICD-9-CM* codes for phenome-wide association studies in the electronic health record. PLoS One. 2017;12(7):e0175508. doi:10.1371/journal.pone.0175508 28686612 PMC5501393

[21] Wu P, Gifford A, Meng X, . Mapping *ICD-10* and *ICD-10-CM* codes to phecodes: workflow development and initial evaluation. JMIR Med Inform. 2019;7(4):e14325. doi:10.2196/14325 31553307 PMC6911227

[22] Mull HJ, Stolzmann KL, Shin MH, Kalver E, Schweizer ML, Branch-Elliman W. Novel method to flag cardiac implantable device infections by integrating text mining with structured data in the Veterans Health Administration’s electronic medical record. JAMA Netw Open. 2020;3(9):e2012264. doi:10.1001/jamanetworkopen.2020.12264 32955571 PMC7506515

[23] Sánchez-Borges M, Ansotegui I, Cox L. World Allergy Organization grading system for systemic allergic reactions: it is time to speak the same language when it comes to allergic reactions. Curr Treat Options Allergy. 2019;6(4):388-395. doi:10.1007/s40521-019-00229-8

[24] Brown SGA. Clinical features and severity grading of anaphylaxis. J Allergy Clin Immunol. 2004;114(2):371-376. doi:10.1016/j.jaci.2004.04.029 15316518

[25] Ring J, Messmer K. Incidence and severity of anaphylactoid reactions to colloid volume substitutes. Lancet. 1977;1(8009):466-469. doi:10.1016/S0140-6736(77)91953-5 65572

[26] Denny JC, Bastarache L, Ritchie MD, . Systematic comparison of phenome-wide association study of electronic medical record data and genome-wide association study data. Nat Biotechnol. 2013;31(12):1102-1110. doi:10.1038/nbt.2749 24270849 PMC3969265

[27] Salkind N. *Encyclopedia of Research Design*. SAGE Publications, Inc.; 2010. doi:10.4135/9781412961288

[28] US Centers for Disease Control and Prevention. Program focus: medication safety program. Accessed August 21, 2022. https://www.cdc.gov/medicationsafety/program_focus_activities.html

[29] Fosnot S, Currier K, Pendell J, Jeffres MN. Comparison of immediate hypersensitivity reactions to preoperative antibiotics in patients labeled as penicillin allergic. Surgery. 2021;170(3):777-782. doi:10.1016/j.surg.2021.02.063 33838879

[30] Sandrowski K, Edelman D, Rivlin M, . A prospective evaluation of adverse reactions to single-dose intravenous antibiotic prophylaxis during outpatient hand surgery. Hand (N Y). 2020;15(1):41-44. doi:10.1177/1558944718787264 30009635 PMC6966299

[31] Goss FR, Lai KH, Topaz M, . A value set for documenting adverse reactions in electronic health records. J Am Med Inform Assoc. 2018;25(6):661-669. doi:10.1093/jamia/ocx139 29253169 PMC6251510

[32] Macy E, Poon K-Y T. Self-reported antibiotic allergy incidence and prevalence: age and sex effects. Am J Med. 2009;122(8):778.e1-778.e7. doi:10.1016/j.amjmed.2009.01.034 19635279

