# Supplementary material for: A Novel Informatics Tool to Detect Periprocedural Antibiotic Allergy Adverse Events for Near Real-time Surveillance to Support Audit and Feedback
Source: JAMA Netw Open. 2023 May 17;6(5):e2313964. doi: 10.1001/jamanetworkopen.2023.13964 (PMC10193175; doi:10.1001/jamanetworkopen.2023.13964)
Supplement: Supplement 1. — eTable 1. Candidate Algorithm Variable Definitions eTable 2. ICD-10-CM Codes and Associated PheCodes for Possible Adverse Reaction to Antibiotic Medication eTable 3. Keywords Used to Search Electronic Health Record Text Notes eTable 4. Iterative Changes to the Algorithm eFigure 1. Sampling Strategy for Training and Testing Data Sets eFigure 2. Receiver Operating Curve for Model [file jamanetwopen-e2313964-s001.pdf]

Supplemental Online Content

Reyes Dassum S, Mull HJ, Golenbock S, et al. A novel informatics tool to detect periprocedural antibiotic allergy adverse events for near real-time surveillance to support audit and feedback. *JAMA Netw Open*. 2023;6(5):e2313964. doi:10.1001/jamanetworkopen.2023.13964

eTable 1. Candidate Algorithm Variable Definitions

eTable 2. *ICD-10-CM* Codes and Associated PheCodes for Possible Adverse Reaction to Antibiotic Medication

eTable 3. Keywords Used to Search Electronic Health Record Text Notes

eTable 4. Iterative Changes to the Algorithm

eFigure 1. Sampling Strategy for Training and Testing Data Sets

eFigure 2. Receiver Operating Curve for Model

This supplemental material has been provided by the authors to give readers additional information about their work.

© 2023 Reyes Dassum S et al. *JAMA Network Open.*

eTable 1. Candidate Algorithm Variable Definitions

Data Element	Definition
Allergy Alert in VA EHR	The first potential indicator of an allergy adverse event was an entry in the VA's Adverse Reaction Tracking (ART) system with an associated antibiotic exposure. This section of the electronic health record (EHR) contains the antibiotic exposure and the resulting reaction and is a structured data element. This entry is labelled as historical (reported reaction) or observed. The 940 available terms for reactions were reviewed (WBE, SRD) and coded as "allergic reaction" e.g., anaphylaxis; "adverse event" e.g., thrombocytopenia; and other/unlikely medication related event (e.g., anger). Discrepancies were then adjudicated through discussion amongst the two clinical reviewers.
Administrative/Coding Data	The second potential indicator of an allergy adverse events included Diagnosis codes from the *International Statistical Classification of Diseases and Related Health Problems, Tenth Revision, Clinical Modification (ICD-10-CM)* for allergy to an antibiotic (ie. Z88.0 Allergy to penicillin), symptoms of allergic reaction (ie. Hives) or adverse reactions. Full list in **Supplementary Table 2**.
Medication Dispensing Data	Dispensed medications. A list of predetermined medications commonly used for treatment of antibiotic-related adverse events (e.g., prednisone, diphenhydramine, intravenous or intramuscular epinephrine). **Full list in Supplementary Table 3**
Text Note Searches	Text note searches for clinical notes containing prespecified keywords for allergy symptom and commonly used treatments for antibiotic-related adverse events (e.g., prednisone, diphenhydramine). Keywords were predetermined and based on prior literature search[2], for common terms describing antibiotic-related allergies or hypersensitivity reactions e.g., hives, rash, anaphylaxis. Full list in **Supplementary Table** 3.

© 2023 Reyes Dassum S et al. *JAMA Network Open.*

eTable 2. *ICD-10-CM* Codes and Associated PheCodes for Possible Adverse Reaction to Antibiotic Medication

ICD-10-CM Code	ICD-10-CM Description	PheCode	PheCode Description
R21.	Rash and other nonspecific skin eruption	687.1	Rash and other nonspecific skin eruption
L27.0	Generalized skin eruption due to drugs and medicaments taken internally	939	Atopic/contact dermatitis due to other or unspecified
L27.1	Localized skin eruption due to drugs and medicaments taken internally	939	Atopic/contact dermatitis due to other or unspecified
T78.2XXA	Anaphylactic shock, unspecified, initial encounter	946	Anaphylactic shock NOS
L50.0	Allergic urticaria	947	Urticaria
L50.9	Urticaria, unspecified	947	Urticaria
T78.3XXA	Angioneurotic edema, initial encounter	949	Allergies, other
T78.40XA	Allergy, unspecified, initial encounter	949	Allergies, other
T78.49XA	Other allergy, initial encounter	949	Allergies, other
T36.1X5A	Adverse effect of cephalosporins and other beta-lactam antibiotics, initial encounter	960.1	Adverse effects of antibacterials (not penicillins)
T36.3X5A	Adverse effect of macrolides, initial encounter	960.1	Adverse effects of antibacterials (not penicillins)
T36.4X5A	Adverse effect of tetracyclines, initial encounter	960.1	Adverse effects of antibacterials (not penicillins)
T36.5X5A	Adverse effect of aminoglycosides, initial encounter	960.1	Adverse effects of antibacterials (not penicillins)
T36.8X5A	Adverse effect of other systemic antibiotics, initial encounter	960.1	Adverse effects of antibacterials (not penicillins)
T36.95XA	Adverse effect of unspecified systemic antibiotic, initial encounter	960.1	Adverse effects of antibacterials (not penicillins)
T36.0X5A	Adverse effect of penicillins, initial encounter	960.2	Allergy/adverse effect of penicillin
T50.905A	Adverse effect of unspecified drugs, medicaments and biological substances, initial encounter	979	Adverse drug events and drug allergies

© 2023 Reyes Dassum S et al. *JAMA Network Open.*

eTable 3. Keywords Used to Search Electronic Health Record Text Notes

Antibiotics	Medications	Symptoms
(("antibiotic") or ("antimicrobial") or ("abx") or ("beta-lactam") or ("cefazolin") or ("vancomycin") or ("cefepime") or ("piptazo") or ("clindamycin") or ("dicloxicillin") or ("diclox") or ("ertapenem") or ("imipenem") or ("mero") or ("meropenem") or ("linezolid") or ("ceftriaxone") or ("nafcillin") or ("daptomycin") or ("ciprofloxacin") or ("levofloxacin") or ("doxycycline") or ("cefuroxime") or ("erta") or ("imi") or ("pip/tazo") or ("tazobactam") or ("ancef") or ("Cefacidal") or ("Cefamezin") or ("Cefrina") or ("Elzogram") or ("Faxilen") or ("Gramaxin") or ("Kefzol") or ("Kefol") or ("Kefzolan") or ("Kezolin") or ("Novaporin") or ("Reflin") or ("Zinol") or ("Zolicef") or ("vanco") or ("clinda") or ("cleocin") or ("pipericillin") or ("zosyn") or ("vancocin"))	(("benadryl") or ("diphenhydramine") or ("prednisone") or ("prednisolone") or ("solumedrol") or ("medrol") or ("epinephrine") or ("epi-pen") or ("epipen") or ("hydroxyzine") or ("vistaril") or ("atarax"))	(("itch*") or ("rash") or ("anaphylaxis") or ("hives") or ("swollen tongue") or ("tongue swelling") or ("throat clos*") or ("wheezing"))

Observations were flagged when ≥1 keyword from each category returned a note from the same date.

© 2023 Reyes Dassum S et al. *JAMA Network Open.*

eTable 4. Iterative Changes to the Algorithm

Data Element	Adjustments
Key word searches in clinical notes	• Adding or removing text note keywords (e.g. adding "wheez*"<br>• Removing drug names with "PM")
Adjusting date-time stamps used for measurement	• Changing the *record entry* date-time to the *episode begin* date-time
ICD-10 Codes	• Conversion to use of PheCodes corresponding to allergy diagnosis and symptoms, rather than individual ICD-10 codes[20,21,26]<br>• Removing codes specified as "historical"
Medication Flag	• Flagging only newly-initiated potential treatments (e.g. a steroid order was not flagged if steroids had also been ordered in the 90-days prior<br>• Creation of a combined medication flag (for steroid, antihistamine, epinephrine, and combinations)
Surveillance Window	• Reduced from 45 to 10 days given lack of specificity between the event occurrence and the exposure with longer surveillance periods.

© 2023 Reyes Dassum S et al. *JAMA Network Open.*

eFigure 1. Sampling Strategy for Training and Testing Data Sets

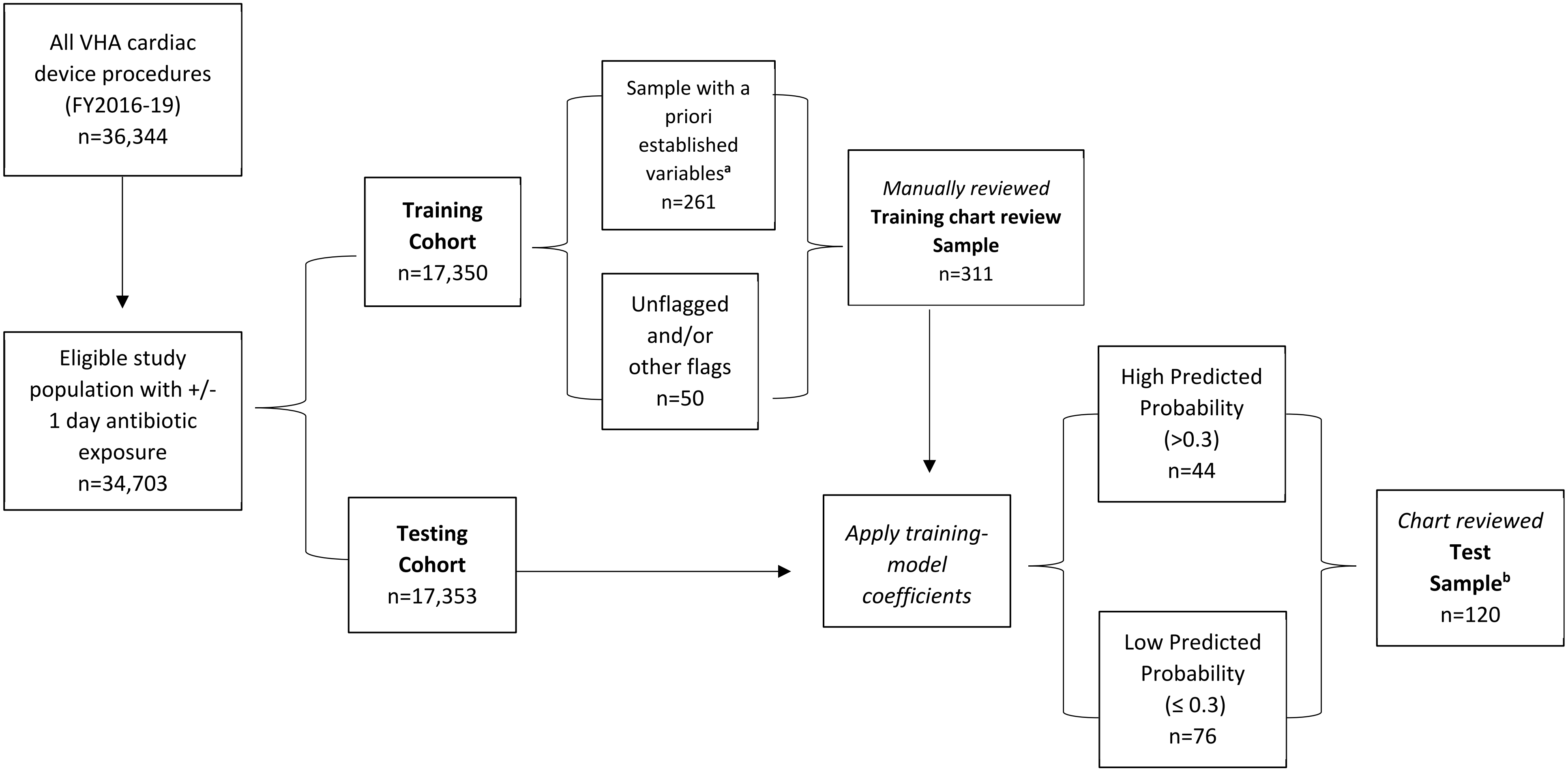

[a]Training sample includes any patient record with one or more flags: VA Allergic Reaction Tracking (ART) system entry, ICD-10-CM code for potential antibiotic-related allergy, administered diphenhydramine and steroid within (+/-) 1 day, administered epinephrine.

© 2023 Reyes Dassum S et al. *JAMA Network Open.*

Training sample also includes a random sample of 50 patient records where: 1) No flag was present (27), 2) Keyword detected in clinical note, diphenhydramine without steroids, and/or steroids without diphenhydramine.

[b]To ensure adequate sampling of true positive cases, a split of 80 high probability (predicted probability > 30%) and 40 low probability (predicted probability ≤ 30%) was randomly selected for the test cohort. This planned 80/40 split was 44/76 in the training cohort, due to re-classification of 8 allergic-type reactions that occurred after the random sample was identified.

© 2023 Reyes Dassum S et al. *JAMA Network Open.*

eFigure 2. Receiver Operating Curve for Model

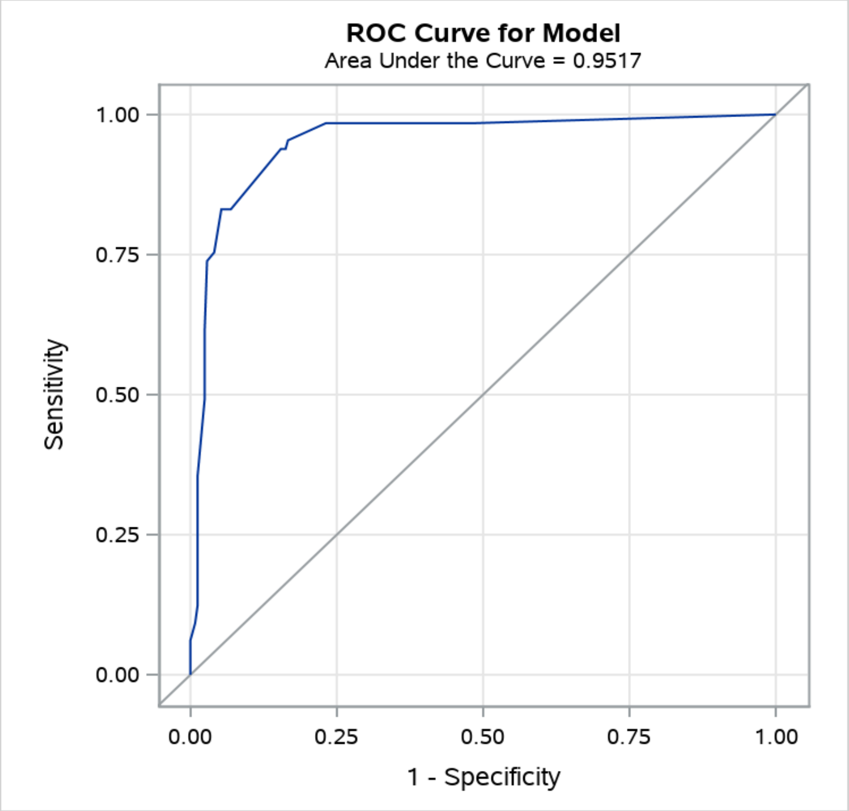

© 2023 Reyes Dassum S et al. *JAMA Network Open.*
