# Supplementary material for: A Novel Informatics Tool to Detect Periprocedural Antibiotic Allergy Adverse Events for Near Real-time Surveillance to Support Audit and Feedback
Source: JAMA Netw Open. 2023 May 17;6(5):e2313964. doi: 10.1001/jamanetworkopen.2023.13964 (PMC10193175; doi:10.1001/jamanetworkopen.2023.13964)
Supplement: Supplement 2. — Data Sharing Statement [file jamanetwopen-e2313964-s002.pdf]

Data Sharing Statement

Reyes Dassum. A Novel Informatics Tool to Detect Periprocedural Antibiotic Allergy Adverse Events for Near Real-time Surveillance to Support Audit and Feedback. *JAMA Netw Open*. Published May 17, 2023. doi:10.1001/jamanetworkopen.2023.13964

Data
Data available: No

Additional Information
Explanation for why data not available: “VA data will be made available to VA researchers via a data use agreement. Code underlying results will be made available via outreach to the corresponding author.”
